# Colour maps for representation of quantitative metrics in radiology: meaningful or misleading?

**DOI:** 10.1186/s13244-026-02414-1

**Published:** 2026-10-12

**Authors:** Nico Sollmann, Maria T. Wetscherek, Barbara D. Wichtmann, Miha Fuderer, Clemens C. Cyran, Edwin H. G. Oei, Fabio Crameri, Nandita M. deSouza

**Affiliations:** 1 Department of Diagnostic and Interventional Radiology, University Hospital Ulm, Ulm, Germany; 2 Department of Nuclear Medicine, University Hospital Ulm, Ulm, Germany; 3 Department of Diagnostic and Interventional Neuroradiology, School of Medicine and Health, Technical University of Munich, TUM Klinikum Rechts der Isar, Munich, Germany; 4 TUM-Neuroimaging Center, TUM Klinikum Rechts der Isar, Technical University of Munich, Munich, Germany; 5 Department of Radiology, University of Cambridge and Cambridge University Hospitals, NHS Foundation Trust, Cambridge, UK; 6 Clinic of Neuroradiology, University Hospital Bonn, Bonn, Germany; 7 German Center for Neurodegenerative Diseases (DZNE), Bonn, Germany; 8 Computational Imaging Group for MR Diagnostics & Therapy, Center for Image Sciences, UMC Utrecht, Utrecht, The Netherlands; 9 Department of Radiology, LMU University Hospital, LMU Munich, Munich, Germany; 10 Department of Radiology and Nuclear Medicine, Erasmus MC University Medical Center Rotterdam, Rotterdam, The Netherlands; 11 Undertone.design, Bern, Switzerland; 12 Division of Radiotherapy and Imaging, The Institute of Cancer Research, London, UK

**Keywords:** Colour palettes, Biomarkers, Cardiac, Musculoskeletal, Neurocognitive

## Abstract

**Abstract:**

The increased utility of quantitative metrics in medical imaging has prompted the use of colour representation of the data. Colour schemes for image display can highlight pathology and distinguish normal anatomy, thus potentially aiding image interpretation. Adding colour schemes to images may serve the sole purpose of visualising an abnormality or may seek to represent and meaningfully convey a quantitative metric. An early use of colour representation is exemplified by colour Doppler in ultrasound (US), where a two-sided linear scale is commonly applied. More recently, many schemes have arisen, ranging from a single colour to depict a segmented region to a range of colours that embrace the entire colour spectrum. These may be applied as continuous or discrete palettes, depending on the software vendor or even on individual user settings. The lack of standardisation of colour schemes across the medical community, especially in medical imaging, risks misinterpretation of quantitative data and potential unintended negative clinical consequences. The purpose of this article is to review the colour schemes used for quantitative imaging across US, computed tomography (CT), magnetic resonance imaging (MRI), and positron emission tomography (PET), the way in which they are applied, the clinical applications where their use is desirable for enhanced interpretation, and the pitfalls where standardisation is lacking in a variety of clinical applications. Finally, we discuss some solutions that could be employed to enable standardisation and augment the use of colour in image interpretation.

**Key Points:**

***Question*** Colour schemes in medical imaging can highlight pathology, but standardisation is lacking for most imaging modalities, thus risking misinterpretation of quantitative data.

***Findings*** For T1, T2, and T2* maps and their inverses, colour map recommendations have recently been proposed, but are mostly lacking for other quantitative data.

***Critical relevance statement*** Standardised colour maps can enhance data analyses across scanners and institutions, which is important for multicentre studies and handling big data. Furthermore, standardised and harmonised colour maps could improve lesion conspicuity, cross-platform comparability, and longitudinal reliability.

**Graphical Abstract:**

**Figure:**
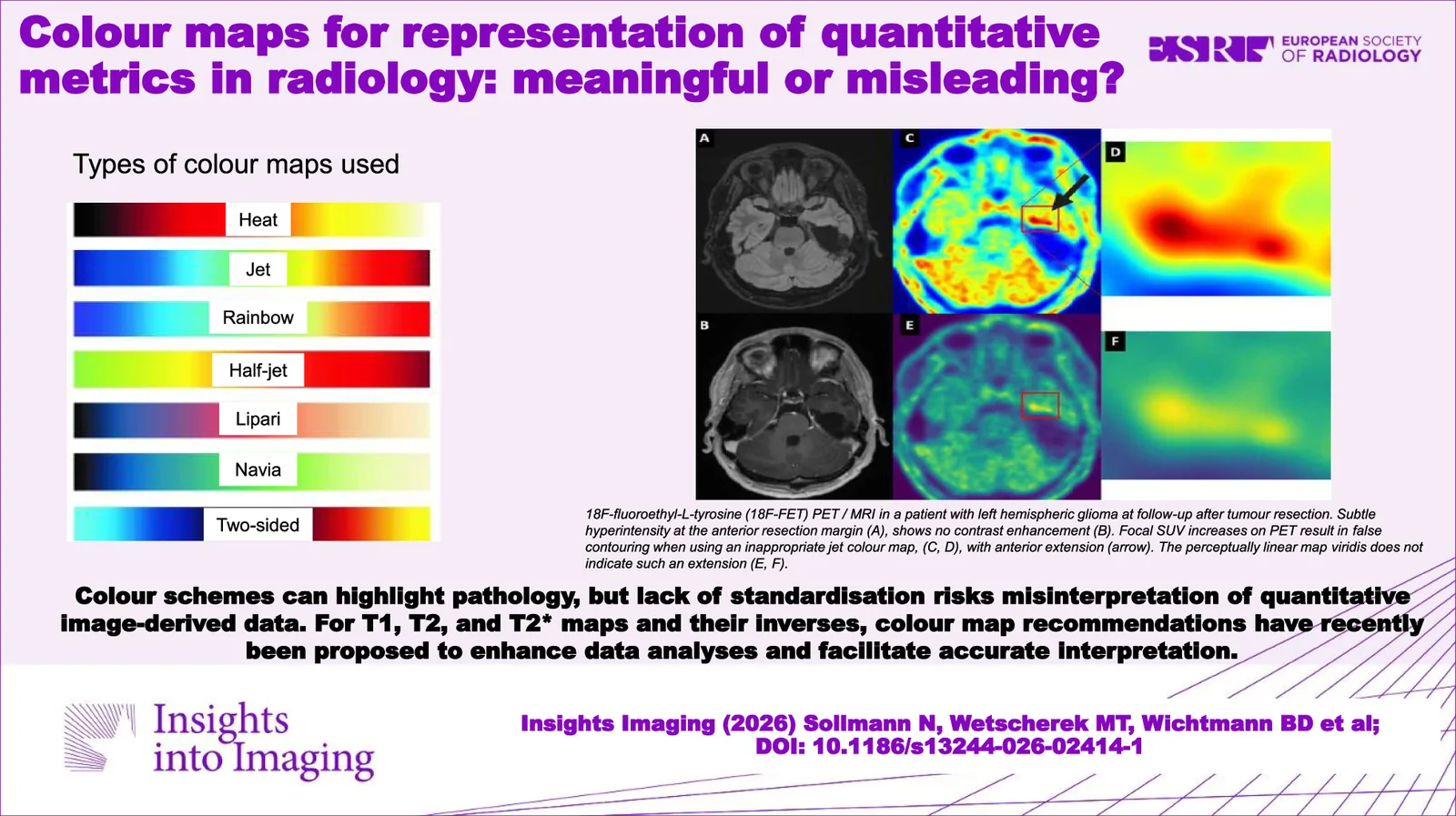

## Introduction

Interpretation of medical images relies on human vision and demands the discrimination of anatomical structures and the effective assessment of pathology. Colour representation of images enhances the transmission of visual information and potentially aids image interpretation. A coloured medical image is more likely to be remembered, compared, and communicated than a pure greyscale image. In addition to luminosity, as evidenced by greyscale images, colour schemes can represent properties of the imaged tissues through hue and chromaticity. The choice of colours can range from a single colour to depict a segmented region to a full range across the colour spectrum. Various colour schemes are employed, often without regard to standardisation, and rely on the availability of software tools and local expertise, so that the interpretation of the resulting colours critically depends on local user familiarity. Moreover, the lack of standardisation across hardware and software systems poses difficulties for longitudinal comparisons when threshold values designating abnormality are revised or when the interpreter has red/green colour blindness. Extracted quantitative parameters that are assigned colours based on their values therefore require that the colour schemes be duly considered and standardised to aid reader understanding and avoid misinterpretation. This critical review focuses on the types of colour representation commonly used in medical imaging modalities in a range of clinical scenarios, and it describes their added value and pitfalls when representing specific diseases.

## Colour scheme representation

**Fig. 1:**
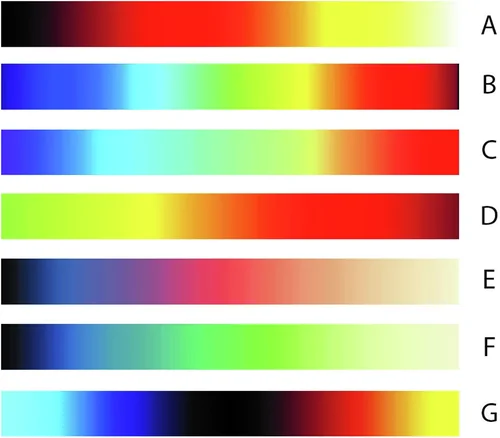
Frequently used colour maps: heat map (**A**), jet (**B**), rainbow (**C**), half-jet (**D**, typically used when low values should map on green and high values map on red (“pathology”), in analogy to traffic lights), colourblind-friendly Lipari map (**E**, prescribed for T1 maps from magnetic resonance imaging (MRI)), colourblind-friendly Navia map (**F**, prescribed for T2 and T2* maps from MRI), and two-sided map for Doppler ultrasound (US, **G**, “BART” map)

The use of colour in radiology may be broadly classified as categorical (i.e., using a very limited set of different colours) or linear (i.e., a one-dimensional property, like velocity or T2, maps to a range of colours) (Fig. 1), although combinations or hybrids of these categories exist (e.g., for multi-parametric use) [1]. In categorical use, typically a single or a few colours are involved, without any specific ordering of colours, so that the colours require labels and annotations to indicate what they depict. Categorical use of colour can be applied to all image types (i.e., quantitative or semi-quantitative imaging and across modalities).

The linear (or continuous sequential) colour scale consists of a continuously varying colour scheme over a range of values within a tissue of interest. Popular choices for this colour scheme are the heat scale (where the colour mimics the emission of a glowing object, going from black through red to yellow), a rainbow scale, or a jet scale. Linear continuous scales are used for data with quantitative meaning and represent the distribution of quantitative values within the image by mapping ranges of values to specific colours. Quantitative parameters from a variety of imaging modalities are often displayed in this way: shear and stiffness on ultrasound (US), perfusion metrics on computed tomography (CT) or magnetic resonance imaging (MRI), relaxometry parameters, MR proton-density fat fraction (PDFF), stiffness on MR elastography, metabolite ratios on MR spectroscopic imaging, maximum standardised uptake value (SUVmax) on PET, and many others. Variants of the linear scale are the two-sided linear scale, for example in Doppler US, where two different linear scales are used for the two directions of flow (Table 1). Many other hybrid uses of the linear scale exist, such as overlaying a region of a linearly-coloured quantitative map onto a grey-valued anatomical image, or using colour in dual-window CT to more easily distinguish the two windows.

**Table 1:** Clinically available biomarkers and selection of the advantages and pitfalls of their colour representations

Tissue property/biomarker	Imaging modality	Colour scheme	Common clinical applications	Selection of advantages/pitfalls
Blood flow (velocity, direction)	Doppler US	Bi-linear discretised	Arterial/venous flow in the lower/upper limbs, abdominal organs, and neck vessels	Standardised presentation
Perfusion (*K*^trans^, *K*_ep_, *V*_e_, rCBV, rCBF)	DCE-MRI, DSC-MRI, ASL	Linear rainbow, heat map, Brewer and others	Tumour perfusion, cardiac perfusion, and stroke imaging	No standards, often no colour bars used
Metabolism (SUV_max_, SUV_min_)	^18^FDG-PET/CT, ^99m^Tc SPECT	Linear rainbow, heat map, and others	Neurodegenerative disorders, tumour metabolism, and metastatic disease	Standardised presentation, but without distinct scales
Relaxivity (T1, T2, T2*)	MRI	Linear rainbow, heat map, and others (Lipari and Navia maps recommended)	Cartilage, tumour hypoxia, and cardiac imaging	Standardised colour maps are available and recommended
Tissue elasticity (stiffness, kPa)	US, MRI	Linear rainbow and others	Tumour (mostly breast), liver imaging	Mostly non-standardised, depending on the vendor
Fat content (PDFF, UDFF, %)	US, MRI	Linear rainbow and others	Liver imaging, bone marrow, musculature	Standardised regarding endpoints (0–100%), but no universally used scheme
DTI (FA and others)	MRI	R/G/B	Tract and microstructure imaging (tumour, neurotrauma, and others)	Standardised regarding direction encoding
CEST (APT)	MRI	Linear rainbow	Tumour imaging (mostly brain)	Standardised presentation (tumour regions mostly depicted in red, healthy regions mostly in green)
MRS (metabolite ratios)	MRI	Arbitrarily colour-coded for metabolites of interest	Tumour detection, response assessment	Highlights abnormal metabolite ratios (based on local reference values)

Between categorical and linear colour representations, a discretised (or stratified) colour scale can be used, predominantly to display the risk of pathology or malignancy. Discretised colour scales based on threshold values or ranges also lend visual impact to the depiction of pathology. Here, one or two bright colours draw attention to the abnormality against a dark or contrasting background, and again, the choice of colour is by individual preference. Typically, a part of the rainbow scale is used for this purpose (e.g., red (R)/green (G)/blue (B)), analogous to a traffic light, with green indicating normal and red indicating an abnormal or pathologic finding. This scale is not always discretised; for example, in amide proton transfer (APT)-MRI, a continuous scale from 0% (green) to 5% (red) is typically recommended [2]. Furthermore, those scales risk ignoring the true quantitative data in the image and thus can introduce operator bias during image processing.

**Fig. 2:**
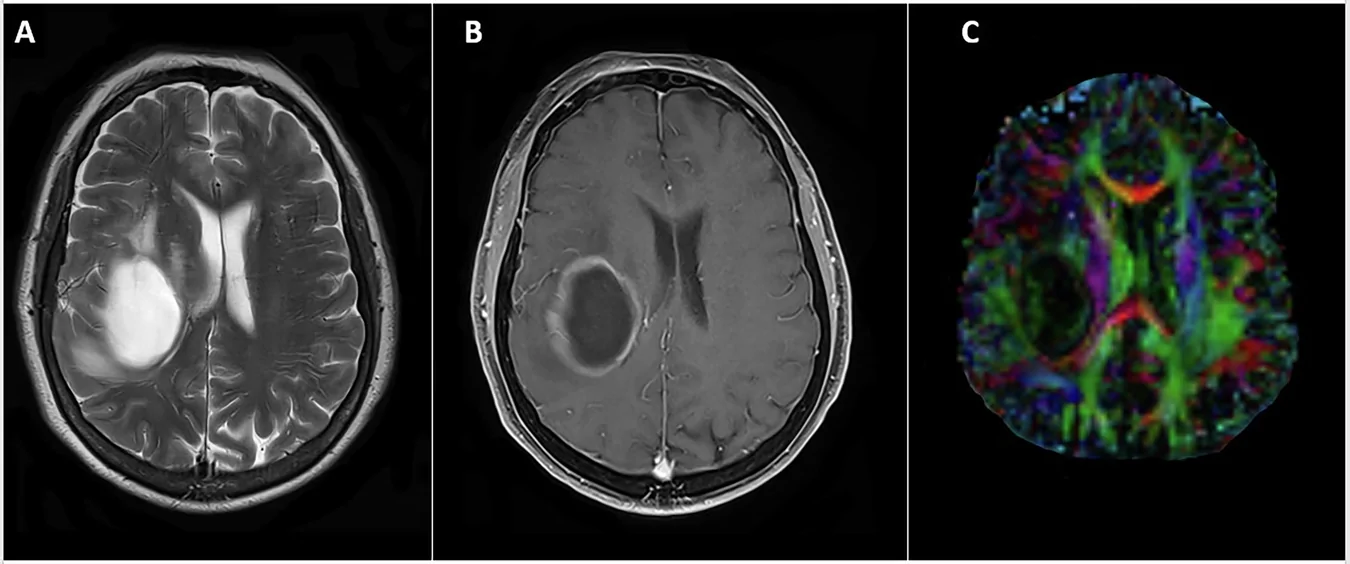
Diffusion tensor imaging (DTI) in a patient with high-grade glioma as displayed on axial standard T2-weighted imaging (**A**) and contrast-enhanced T1-weighted imaging (**B**), with DTI showing directional colour coding of white matter tracts (**C**). According to directionally encoded colour, red, green, and blue (R/G/B) depict a principal direction (left–right, anterior–posterior, and superior–inferior), and the tumour seems to compress and disrupt white matter tracts (**C**)

Another variant of colour representation is multi-parametric, where all combinations of luminance, saturation, and hue (or of R/G/B) can appear in the image, since it shows multiple elements of the property at once. In diffusion tensor imaging (DTI), for example, where redness indicates an anisotropy in the left-right, green for anterior-posterior, and blue for superior-inferior direction, a saturated purple would then indicate an oblique orientation (Fig. 2) [3].

To date, the use of quantitative imaging biomarkers in clinical decision-making remains limited. Thresholds separating abnormal from normal values are poorly established. Therefore, biomarker ranges are often used to designate disease categories. Nevertheless, the drive to standardise imaging biomarkers, automation in analysis, and increasing use of artificial intelligence (AI) tools mean that validated quantitative imaging biomarkers will be increasingly exploited for clinical decision-making in the future, so that their representation in colour needs to ensure an appropriate colour scale across the range of values in order to optimally define disease.

## Added value of colour and pitfalls in interpretation for clinical applications

### Tumour imaging

**Fig. 3:**
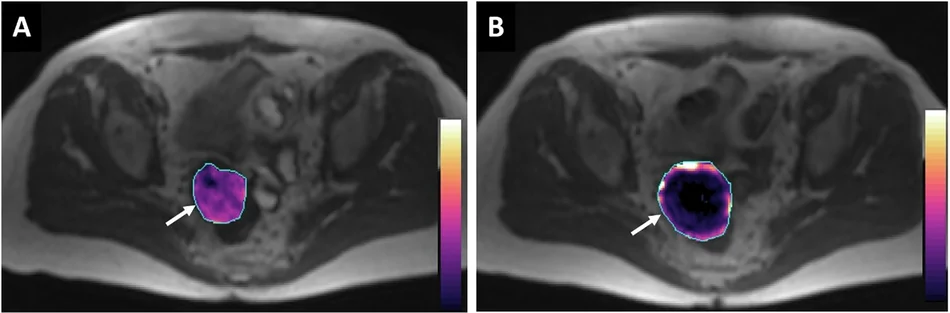
Metastasis from poorly differentiated ovarian cancer (white arrow), showing colour representation of quantitative volume transfer constant (K^trans^) measurements overlaid on T1-weighted axial images before (**A**) and 7 days after treatment (**B**) with an antiangiogenic agent. The colour scale represents K^trans^ values in ovarian cancer that are typically between 0 and 0.25 min^-1^ but are not included on the image, so there is no information about the range of values represented. In **B**, there is a marked reduction in K^trans^ within the tumour, although its size is largely unchanged

Quantitative tumour biomarkers routinely use colour maps to highlight abnormal values of the measured biomarker. Perfusion imaging on Doppler US uses divergent continuous colour scales (e.g., shades of red for one direction and shades of blue for the opposite direction) and has adopted common standards of representation, thereby enabling multicentre studies [4]. CT and MRI have now superseded US in soft tissue tumour diagnosis by providing metrics such as blood flow, flow velocity, permeability, and blood volume. For example, brain MRI exploits relative cerebral blood volume (rCBV), commonly displayed by rainbow colour schemes, for tumour detection and classification [5]. However, the lack of appropriate colour scales to indicate the values being depicted is common [6]. In liver imaging, perfusion is useful in differentiating benign from malignant lesions: in an international prospective multicentre trial, quantitative dynamic contrast-enhanced (DCE)-MRI in addition to contrast-enhanced CT altered the management plan in 13% of patients [7]. Specifically, DCE-MRI metrics can be invaluable in assessing treatment response and planning subsequent management. Here, simplified metrics such as percentage peak enhancement with discretised colour displays [8] can be used for visual appreciation in addition to quantitative metrics such as volume transfer constant (K^trans^) displayed with rainbow schemes or a limited colour palette (e.g., Brewer) [9]. Arterial spin labelling (ASL) perfusion techniques, also using discretised displays, have been applied to illustrate brain tumour response to radiotherapy [10]. In other tumour types, the extent of perfusion abnormality informs the radiotherapy planning either in the primary or adjuvant setting, for example using Technetium-99m (99mTc) single-photon emission CT (SPECT) imaging in liver tumours [11]. Importantly, the use of quantitative functional imaging techniques can reduce radiation dose to organs at risk (e.g., normal lung function can be preserved, sparing normal tissue toxicity [12, 13]). By enabling quantification of tumour vascularisation, the biological effects of antiangiogenic therapies, displayed in colour, can be detected before the tumour begins to shrink (Fig. 3) [14–16]. Perfusion metrics with linear rainbow scales have been widely used to differentiate recurrence from post-radiation necrosis in gliomas [17–19]; in breast cancer, measured values may require a logarithmic rainbow scale to assess recurrence [20]. In some instances, a heat map spectrum is used to highlight the response of metastatic disease [21]. Sometimes, the arbitrary use of two or three colours in a threshold-based form without colour bars is used for illustrative purposes alone [22], for instance to demonstrate recurrence [23]. However, assessment of the quantitative data itself and of changes on treatment are crucial [24], and to meaningfully interpret the quantitative data or any changes, standardisation of the colour schemes representing the data is essential. Currently, in tumour imaging, the use of standardised, discretised colour maps for clinical decision-making requires that the relevant quantitative biomarker thresholds are firmly established. Unfortunately, established thresholds are not recognised and are being explored across various clinical scenarios, making it imperative to standardise the use of colour palettes that can be applied and adapted for clinical use.

Where 18F-fluorodeoxyglucose (18F-FDG) PET/CT is used as the primary tumour staging modality (e.g., lung, lymphoma, head and neck cancer), the SUVmax is displayed using a linear colour scale with options of the full rainbow spectrum or a heat map colour spectrum (e.g., from black through red to yellow and white) [25, 26]. Sometimes threshold-based colourisation of abnormally high SUVmax is used, without accompanying colour bars to indicate the values being highlighted [27]. This visually highlights the abnormality, rather than representing the range of quantitative data in colour. Furthermore, 18F-FDG PET/CT is also widely used for recurrence assessment, particularly in highly metabolic tumours like diffuse large B-cell lymphoma (DLBCL) or metastatic adenocarcinomas. In DLBCL, changes in SUVmax determine the management pathway, particularly when potentially curative treatment options become available [28]. Colour depictions for individual clinical evaluation, therefore, sometimes use contouring methods to display regions within a given percentage of SUVmax or SUVmean [29]. This does not allow assessment of the extent to which quantitative values in this region differ from those in neighbouring tissues.

In some tumours (e.g., brain, head and neck cancer), oxygen-enhanced relaxivity maps (T1, T2*) have been used to explore the presence of tumour hypoxia using a linear rainbow scale [30], or a two-colour divergent scale to indicate the changes in the parameter following oxygen delivery (shades of blue as negative values, shades of red as positive values) [31]. Tissue stiffness as a measure of tumour presence and its invasiveness is quantified with US elastography and MR elastography (in kPa) and utilises linear scales across a rainbow spectrum [32], or a rainbow plus a Brewer scheme to represent different parameters [33]. Techniques such as chemical exchange saturation transfer (CEST), MR spectroscopy, and hyperpolarised MRI (with pyruvate/lactate ratios) have all been explored for quantitative representation of tumour metabolism [34]. A recent meta-analysis has suggested that APT-CEST is a promising technique for improving the discrimination of true progression and recurrence from therapy-related changes in gliomas [35], but is not in routine clinical use. The same applies to MR spectroscopic imaging, trialled in brain, breast, and prostate applications, where efforts to incorporate it into the Prostate Imaging Reporting and Data System (PI-RADS) classification were unsuccessful because sufficient evidence of its utility was lacking [36]. Colour representations of the biomarkers in question generally use rainbow or heat map scales, with the same interpretation pitfalls mentioned previously.

### Cardiac imaging

Colour maps have been used for CT evaluation of myocardial perfusion. Accurate localisation and quantification of the transmural extent of ischaemic lesions, which appear hypodense relative to healthy tissue, are important determinants of treatment, complications, and prognosis [37]. However, colour scale interpretation (hot-iron and rainbow) has lower agreement for less conspicuous ischaemic lesions (25–50% of wall thickness) and a tendency to overestimate size (> 50% of myocardial thickness) [37]. More recently, a discretised colour display has been used in AI-supported tools for atherosclerotic plaque segmentation to highlight calcified, non-calcified, and low-attenuation plaques [38].

In cardiac SPECT and PET images, colour maps are generally used to categorise the level of uptake or defect severity, including changes from stress and rest myocardial perfusion [39]. Greyscale, inverted grey, and rainbow are all convenient and accessible, but can have reduced discriminative power for mild to moderate/severe defects [39]. Scales where the transitions between colours are perceived as edges are misleading because they give the false perception of a significant change in counts, even when the change is minimal [40].

**Fig. 4:**
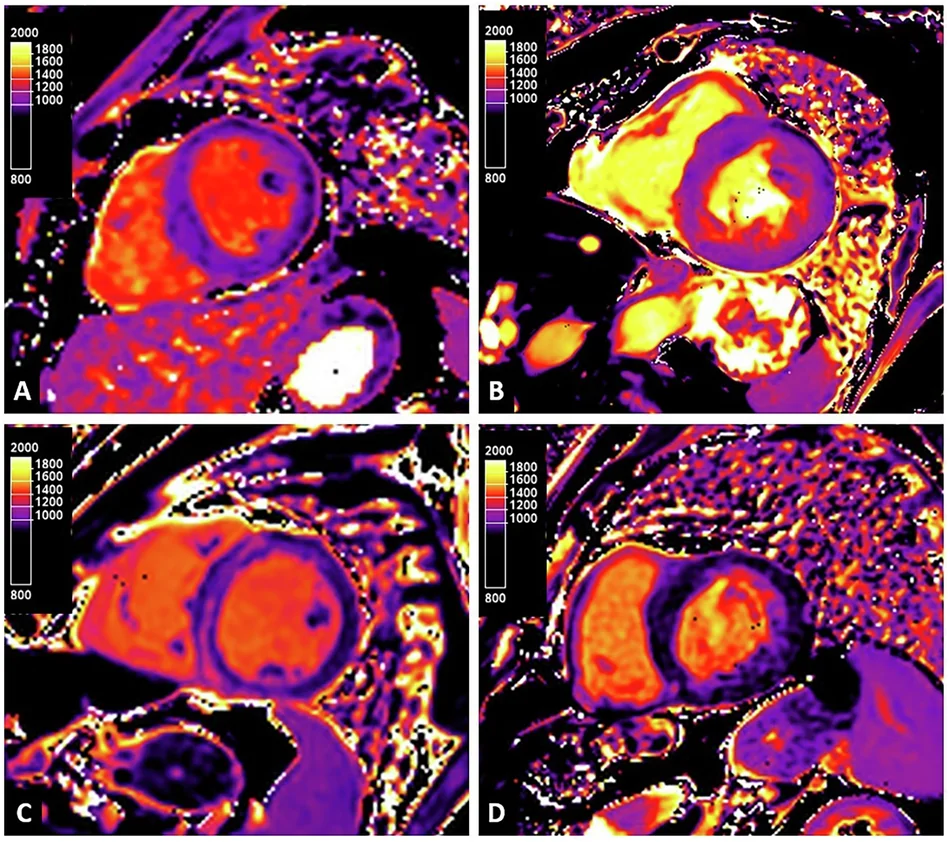
Native myocardial T1 mapping of a normal subject with an overall myocardial T1 relaxation time of 1001 ms (**A**), amyloidosis showing diffuse increase in myocardial T1 relaxation time at 1168 ms (**B**), acute myocarditis secondary to cocaine abuse showing focal mid-myocardial increase in T1 relaxation time at 1065 ms (**C**), and Anderson-Fabry disease showing diffuse decrease in myocardial T1 relaxation time at 874 ms (**D**). Local normal myocardial T1 values at 1.5 T: 960–1043 ms. The range of T1 values represented from 1000 ms to 2000 ms uses five distinguishable colour schemes for each 200 ms increment, while normal myocardial values would be black and difficult to discriminate from the dark purple of 1000 ms to 1200 ms. According to recent recommendations, the Lipari colour scheme could be used for T1 mapping [87]

Myocardial mapping of T1, T2, and T2* relaxation times (in ms) and extracellular volume (ECV, in %) is used to assess focal or diffuse myocardial disease [41, 42]. Such myocardial tissue characterisation has implications for diagnosis and therapeutic decision-making in various cardiac pathologies, including myocardial fibrosis, oedema, ischaemia, and infiltration [41–43]. Specifically, T1, T2, T2*, and ECV mapping generally use continuous non-linear fire or jet colour schemes (Fig. 4), with ranges set at 800–2000 ms, 0–120 ms, 0–50 ms, and 0–100%, respectively, where the assigned colours around the normal reference values are visually indistinguishable, while a clear difference is discernible for values in pathological ranges [42]. The highest native T1 values (> 1200 ms) are in acute and chronic myocardial infarction (localised) and amyloidosis (diffuse), while the lowest are in lipomatous metaplasia/fat (localised, < 900 ms) and Anderson-Fabry disease and iron overload (diffuse, < 950 ms) (Fig. 4) [42]. Colour display with a continuous scale has also been used for visualisation of lung T1 and T2 mapping [44, 45], delayed contrast dynamics [46], or ventilation and perfusion mapping [47, 48]. Colour maps can be used to represent directional information, such as the principal direction of respiratory motion of a surface or volume element. Usually, R/G/B colour schemes are used, where the colours red, green, and blue represent motion along the principal directions [47].

Four-dimensional (4D) flow MRI quantifies blood flow particularly for diagnosis and follow-up assessment of congenital heart disease, valvular heart disease, and congenital and acquired disease of the aorta [49], facilitating the detection and understanding of blood flow alterations in different pathologies [50, 51]. It can be performed using maximum-velocity projections, instantaneous streamlines, and time-resolved path lines, flow paths, flow-velocity vectors, and isosurfaces, colour-coded based on flow speed [49–51]. To avoid vector overlap in three-dimensional (3D) velocity vector representations [50], colour-coded velocity vectors are restricted to cut planes, allowing the colour scale to be adapted to the velocities. For volumetric visualisation, streamlines and particle traces are particularly well suited [52]. Visualisation should include dynamic visualisation of the complete 3D volume. Streamlines and path lines that abruptly change direction or drift out of the lumen can indicate phase wraps or uncompensated background phase offsets [50]. Keeping streamlines short minimises the risk for misinterpretation [51]. To display velocities in 4D flow, typically a jet colour map is used, where the upper limit of the colour scale is determined by the maximum velocity in the displayed anatomy of interest (e.g., 0–110 cm/s to display velocity). The same concept applies to both streamlines and 3D vectors. Discrete colour coding can be used to distinguish particle traces originating from seed points in different vessels and to distinguish the different ventricular flow components (i.e., direct flow, delayed ejection flow, retained inflow, and residual volume). Unfortunately, the limits of the scale vary with individual examinations; to effectively compare flow patterns across cardiac phases, the same limits for the colour map should be used based on the maximum and minimum values found across different cardiac phases and should be standardised [52].

### Neurodegenerative/neurocognitive imaging

**Fig. 5:**
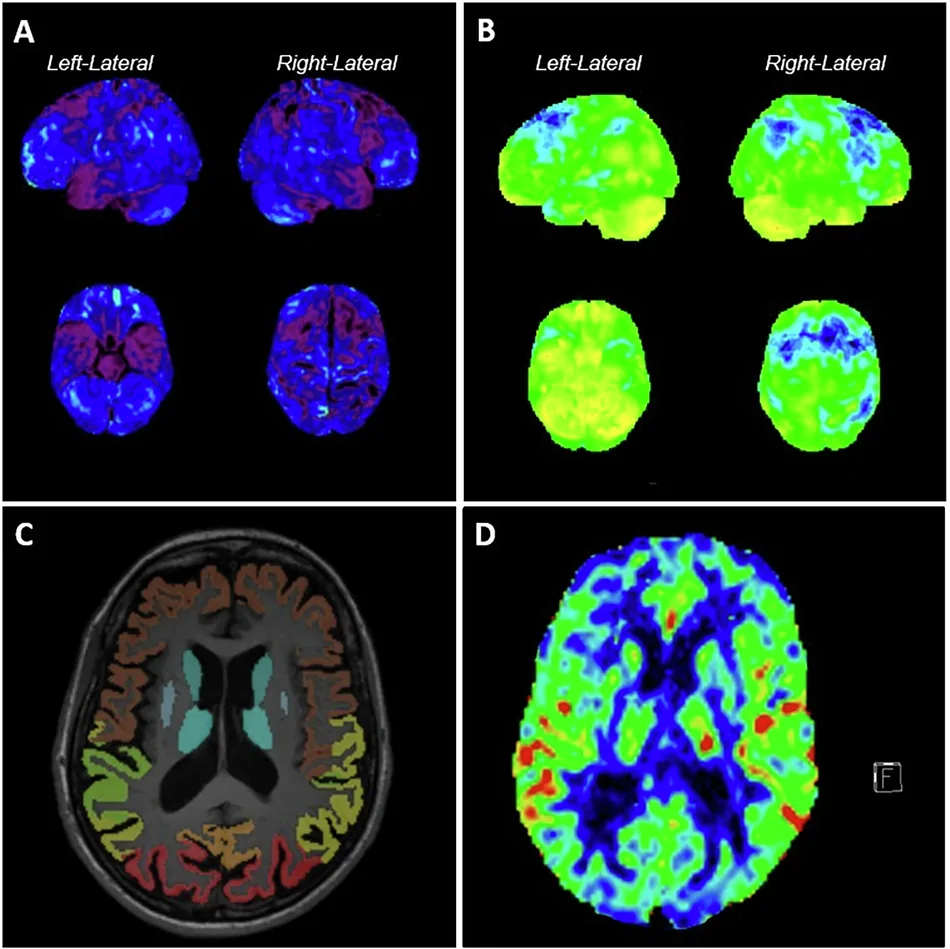
Images from 18F-fluorodeoxyglucose (18F-FDG) positron emission tomography (PET)/magnetic resonance imaging (MRI) in a patient with suspected Alzheimer’s disease (AD) vs. frontotemporal dementia, showing standardised uptake value (SUV)-derived maps on single-subject level (**A**) and in comparison to a healthy control reference cohort (**B**). Areas of hypometabolism when comparing the individual patient to the control reference cohort are shown in blue; in this case, the frontal and parietal areas (**B**). In addition, segmentations on an axial T1-weighted image with arbitrary colour-coded overlays are shown for complementary morphometry with regional brain volume assessments (**C**), and an axial cerebral blood flow (CBF) map with a commonly used jet colour scheme from arterial spin labelling (ASL) in the same patient is displayed (**D**)

Structural MRI interrogating cortical thickness, regional brain volumes (e.g., hippocampal atrophy), morphometry, and surface-based analyses typically use colour overlays to depict regional atrophy or deviation from normative databases, but do not directly represent quantitative metrics (Fig. 5). Resting-state functional networks and task-based activation patterns are represented with colour maps for statistical activation strength (e.g., *z*-scores, correlation coefficients). Work on functional MRI interrogating cognition uses colour to highlight a focus of activity; this is usually a single colour or a hot-iron scheme [53].

In clinical routine, systematic brain imaging is recommended for any first-line cognitive impairment and usually entails MRI with standard sequences to rule out intracranial masses, hydrocephalus, stroke, and/or vascular lesions, which does not require representation with colour maps [54]. In Alzheimer’s disease (AD) and mild cognitive impairment, current evidence from a meta-analysis does not support the routine use of 18F-FDG PET scans in clinical practice [55]. Where 18F-FDG-PET is used, SUV is usually displayed as a rainbow colour scheme [56]. The topographical use of biomarkers derived from MRI, such as perfusion metrics from ASL and cerebral blood flow (CBF), among others, may be useful as complementary parameters (Fig.  5) [57, 58].

Amyloid PET is considered the reference standard for imaging in AD, but its limited availability means it is not routinely used. A range of other molecular markers in PET (including tau protein, inflammatory markers, cholinergic markers, and others) remain under investigation for imaging in dementia and especially AD [59, 60]. All these schemes tend to use linear rainbow scales, with varying representations of the colour [61], so that there is a complete lack of understanding of the significance of any particular colour representation. With the ongoing increase in drug therapies for AD, the need for monitoring response will necessitate rigorous imaging assessments [62], particularly in multicentre settings for rapid drug licensing. Standardisation of colour representations within these quantifiable images will be critical.

### Metabolic/fat imaging

Colour-coded parametric maps may aid clinical interpretation of quantitative inflammation and fat fraction (FF) imaging, in which spatial variations in metabolic activity directly influence diagnosis, risk stratification, and treatment planning. In inflammation, quantitative PET uptake maps and MRI-derived inflammatory indices presented as sequential colour maps (e.g., hot-iron, inferno) can highlight regions of increased activity. Warm-tone colour scales enable localisation of inflammatory foci in rheumatoid arthritis, myocarditis, or vascular inflammation, assessment of disease extent, and monitoring of response to therapy [63]. Diverging colour maps are occasionally applied when both supra- and sub-baseline inflammatory signals are relevant, for example in evaluating immunomodulatory therapy effectiveness or detecting residual inflammation post-treatment.

In FF imaging, colour maps facilitate visual discrimination of lipid content and regional distribution. The FF maps are commonly used in liver or muscle imaging to assess the severity of non-alcoholic fatty liver disease, muscle quality in sarcopenia, and body composition in obesity or metabolic syndromes [64–66]. Specifically, a jet or rainbow colour map has been used to outline PDFF values in liver images before and after treatment, together with corresponding greyscale images to highlight improvements in steatosis [65]. Colour-coded maps assist in segmenting adipose and lean tissue, tracking longitudinal changes in fat distribution, and guiding targeted interventions or preoperative planning in bariatric or metabolic surgery. Accurate interpretation depends on standardised and perceptually uniform colour-value mappings. Non-uniform schemes can exaggerate mid-range differences or mask clinically significant transitions, influencing inter-reader reproducibility.

**Fig. 6:**
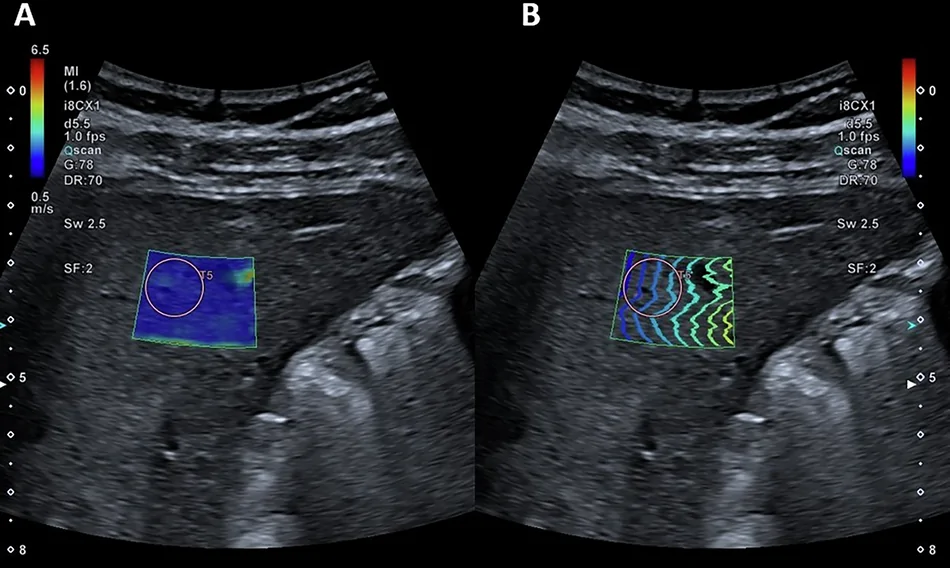
Ultrasound (US) images of the liver overlaid with colour-coded elastography (**A**) and shear-wave elastography (with no clearly defined units for the colour-coded lines shown) (**B**)

To assess metabolic dysfunction-associated steatotic liver disease, B-mode US imaging with greyscale has been used, with hepatic fat content playing a crucial role as a prognostic indicator [67]. The organ is depicted as points of variable brightness, with related wide inter- and intra-observer variability when evaluated purely visually [68]. Quantification can be achieved by combining liver stiffness and elastography assessments (Fig. 6); more recently, US-derived FF (UDFF) evaluations have also been investigated [69, 70]. When displayed in colour, US-derived ranges of UDFF have been shown using jet or rainbow schemes or overlaid on B-mode greyscale images [71, 72].

### Musculoskeletal imaging

Quantitative MRI techniques to assess the composition of the articular cartilage are most often applied in clinical research studies on knee osteoarthritis and include relaxometry techniques (i.e., T2 and T1rho mapping, T1 mapping after the administration of a gadolinium-based contrast agent), as well as techniques that probe the amount of macromolecular content (specifically glycosaminoglycans (GAGs)), such as sodium MRI and GAG-CEST [73]. The associated parametric maps are colour-coded arbitrarily, but commonly areas of healthy cartilage are presented as blue or green, whereas regions of diminished quality are coded in orange or red (e.g., measured as low water content on T2 mapping, representing low content and network integrity of collagen fibres, or low GAG content with other techniques). The issues surrounding the quantitative MRI colour maps for muscle and bone lesions are similar as for cartilage [74, 75].

**Fig. 7:**
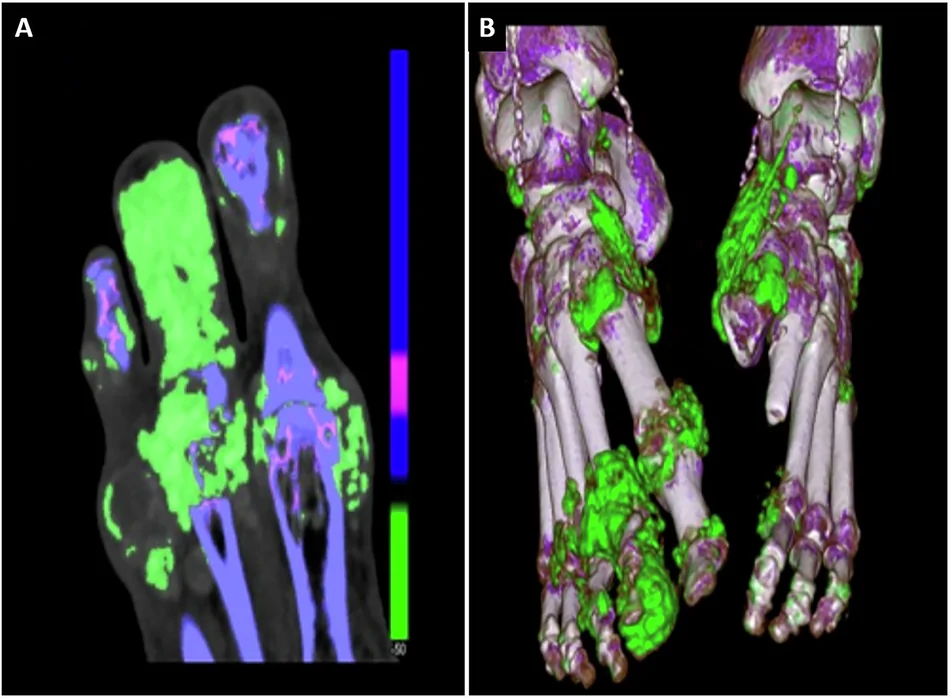
Two-dimensional (2D; **A**) and three-dimensional (3D; **B**) images from dual-energy computed tomography (DECT) of the feet, showing deposition of monosodium urate crystals in gout with categorical colours and surface rendering (green). This does not represent an ultimate quantitative metric

There are several applications in routine clinical musculoskeletal practice that leverage the spectral information provided by dual-energy CT (DECT). Of these, the detection and quantification of monosodium urate crystals in gout, as well as bone marrow oedema by means of material decomposition capabilities of DECT, are the most widespread [76]. For both applications, colour maps indicate the location of crystal deposits or bone marrow superimposed on anatomical CT images (Fig. 7). These maps typically do not represent quantitative values but rather the presence of abnormalities based on thresholding. Colours are defined arbitrarily, with typically green indicating both crystal deposits and areas of bone marrow oedema (Fig. 7).

Doppler US is also frequently used in musculoskeletal imaging [77], particularly for arthritis and soft-tissue tumour assessment, with the same issues and caveats as described above.

## Summary and future directions

**Fig. 8:**
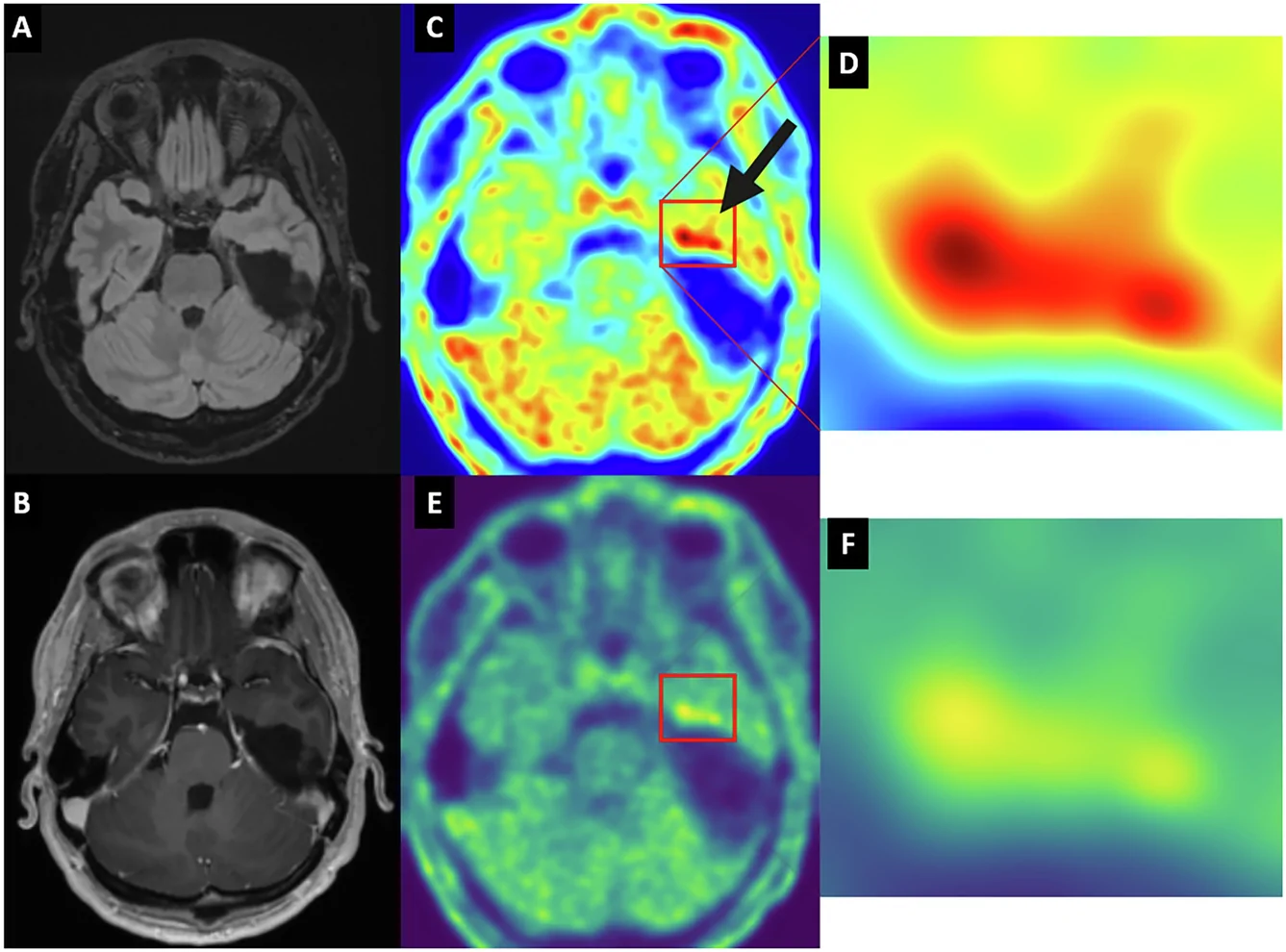
Images from 18F-fluoroethyl-L-tyrosine (18F-FET) positron emission tomography (PET)/magnetic resonance imaging (MRI) in a patient with left-hemispheric glioma after tumour resection and in the stage of follow-up imaging. Imaging by fluid-attenuated inversion recovery (FLAIR) shows only subtle hyperintensity at the anterior resection margin within temporal areas (**A**), while no contrast enhancement was present at this location according to high-resolution contrast-enhanced T1-weighted imaging (**B**). However, at the anterior resection border, focal increases of standardised uptake value (SUV) are suspected, with false contouring by the use of an inappropriate colour map, in this case jet (**C**, **D**), suggesting a cuspated extension based on SUV of the lesion more anteriorly (black arrow). The perceptually linear map (Viridis) is much less indicative of such an extension (**E**, **F**)

While the use of colour for quantitative imaging metrics can enhance interpretation, non-standardised colour maps can lead to unrecognised misrepresentation and misinterpretation (Fig. 8), with potential unintended negative clinical consequences. Unfortunately, these non-standardised colour displays are the norm rather than the exception, and no formal data are available on the extent of unintentional misuse in clinical practice [78]. Although widely used, rainbow or jet colour schemes, in which yellow is the brightest colour, are far from optimal. Optimal colour maps require (1) even colour contrast along the colour map, (2) high overall colour contrast, (3) high overall lightness contrast, (4) intuitive ordering of the colours, and (5) recognisability of the type of information from the colour scheme [1, 79–86].

Standardised colour maps can enhance data analyses across scanners and institutions, which is important for multicentre studies and handling big data. Emerging AI-optimised colour maps could facilitate computational feature extraction while maintaining radiologists’ interpretability, thereby enabling the integration of human- and machine-assisted evaluation in research and clinical workflows. Hence, standardised and harmonised colour maps could improve lesion conspicuity, cross-platform comparability, and longitudinal reliability. Ultimately, colour maps should facilitate accurate understanding and interpretation of the underlying data, so that there is little to no room for mistakes or misunderstandings resulting from the application of colour schemes to the original data.

Recently, the Quantitative MR Group of the International Society of Magnetic Resonance in Medicine (ISMRM) has made efforts to standardise quantitative MRI colour maps for T1, T2, and T2* maps and their inverses [87]. To generate an internationally agreed-upon consensus recommendation for colour displays of MR relaxometry, a Delphi process was used [88]: the logarithm-processed Navia colour map for displaying T2, R2, T2*, and R2*, and the logarithm-processed Lipari colour map for displaying T1 and R1 data are recommended [86, 87]. Although tailored to MR relaxometry data, this consensus recommendation might be adopted more widely [86, 87]. In fields where more modality-specific colour map recommendations may be required, this initiative could trigger comparable international efforts.

## Data Availability

Not applicable.

## Abbreviations

18F-FDG: 18F-fluorodeoxyglucose
18F-FET: 18F-fluoroethyl-L-tyrosine
2D: Two-dimensional
3D: Three-dimensional
4D: Four-dimensional
99mTc: Technetium-99m
AD: Alzheimer’s disease
AI: Artificial intelligence
APT: Amide proton transfer
ASL: Arterial spin labelling
CEST: Chemical exchange saturation transfer
CT: Computed tomography
DCE: Dynamic contrast-enhanced
DECT: Dual-energy computed tomography
DTI: Diffusion tensor imaging
FF: Fat fraction
GAG: Glycosaminoclycan
K^trans^: Volume transfer constant
MRI: Magnetic resonance imaging
PDFF: Proton-density fat fraction
R/G/B: Red, green, blue
SPECT: Single-photon emission computed tomography
SUV: Standardised uptake value
UDFF: Ultrasound-derived fat fraction
US: Ultrasound
